# RE: Parity of esteem within the biopsychosocial model: is psychiatry still a psychological profession?

**DOI:** 10.1192/bjb.2024.5

**Published:** 2024-04

**Authors:** Mustafa Alachkar

**Affiliations:** Consultant Psychiatrist and Medical Psychotherapist, Mersey Care NHS Foundation Trust, UK. Email: malachkar@hotmail.com

*The bio-bio-bio model of psychiatry and the psychiatrist's contribution*

I read with great interest the article by Dr O'Reilly and colleagues on parity of esteem within the biopsychosocial model of psychiatry. I strongly echo the authors’ assertion that psychiatry has moved away from being a psychological profession and has adopted an increasingly biomedical model of care, to the neglect of the psychological and social aspects of people's distress. My view is that this is almost so obvious that we need to move beyond highlighting it to thinking about how we can change it. More on this later.

The authors rightly emphasise the value of formulation as a way of understanding our patients and helping them make meaning and connections in their stories. The authors also raise concerns that psychiatric trainees are losing their skills in formulation as they focus more on information-gathering with a view of eliciting symptoms and arriving at a ‘diagnosis’, in keeping with a biomedical model of psychiatry. My worry, however, is that even when formulation is taught and presented, in case presentations at the local teaching for example, it is often presented as a table, claiming to reflect a biopsychosocial model of understanding and containing many pieces of information about the patient that are not connected to one another and that see the patient in fragments, rather than as a multi-layered and relational entity, with a narrative, that exists within a complex political, cultural and socioeconomic context. Presenting a formulation does not, in its own right, mean that the doctor is thinking psychologically about their patient.

I strongly agree with the authors’ argument that psychiatrists at all levels need to be equipped and trained to deliver psychological treatment and not just to refer for it. The authors argue that this is not happening because of ‘the shifting perception of what psychiatrists do – assessing, diagnosing, prescribing, applying the Mental Health Act, and managing and holding responsibility for risk’. I certainly agree with that, but I wonder also whether this perception of the psychiatrist's role serves a function for the psychiatrist him/herself in keeping him/her at a distance from the patient and preventing real connection with the patient and his or her true experience and pain. It is a much more comfortable position, for the psychiatrist, if his/her job is to diagnose, prescribe, refer and make a medical recommendation for detention than if it is to try to truly connect with the deep and painful nature of the patient's distress. Medicalising, the main tool in the predominant ‘bio-bio-bio’ approach of psychiatry, is therefore a defence for the psychiatrist to save him or her from true empathy.

Medicalising (seen here in contrast to thinking psychologically and delivering psychological treatment) is also a defence that helps the psychiatrist to continue to assume the position of the healthy one who is clearly separated from his or her – by definition – sick patient. At the end of the day, interpersonal dynamics, defence mechanisms and human experiences, as may be described in a formulation, are universal and shared by all of us, whereas diagnostic labels are designed in such a way as to – or in order to – keep apart those of us who are seen as ill from those who are ‘healthy’.

To sum up this point, my argument is that psychiatrists themselves drive this heavily biomedical approach to mental health help, as it helps them stay safe, psychologically speaking, and separates them from their patients’ pain and suffering. Adopting the biomedical model of psychiatry also helps psychiatrists to remain aligned with the wider medical profession that they have invested several years training in and that affords them a position of authority and privilege in society that is hard to relinquish. Being a medical professional may provide psychiatrists with a false sense of certainty about their supposedly evidence-based treatment approaches. It may also move them away from their duty to address other needs that the patient may present with that are stronger determinants of their well-being, or lack of, such as inequality, discrimination, marginalisation and other socioeconomic factors. I wonder whether many psychiatrists do not see addressing these issues as part of their responsibility towards their patients, even when they believe they are using a biopsychosocial model of care.

Finally, it may be that the original article is addressed to a different readership from the one that my response is addressed to. This is reflected in the ‘Next steps’ suggested by the authors, which are all valuable suggestions to move forward. However, most of these steps are changes that need to be initiated by the ‘system’, exemplified by the College, NHS trusts or medical education bodies. I believe that change needs to start from psychiatrists themselves, who need to rethink their role and their professional identity. It is this that we, as psychiatrists, have let slip.

